# Radiomic Features From Multi-Parameter MRI Combined With Clinical Parameters Predict Molecular Subgroups in Patients With Medulloblastoma

**DOI:** 10.3389/fonc.2020.558162

**Published:** 2020-10-02

**Authors:** Jing Yan, Lei Liu, Weiwei Wang, Yuanshen Zhao, Kay Ka-Wai Li, Ke Li, Li Wang, Binke Yuan, Haiyang Geng, Shenghai Zhang, Zhen Liu, Wenchao Duan, Yunbo Zhan, Dongling Pei, Haibiao Zhao, Tao Sun, Chen Sun, Wenqing Wang, Xuanke Hong, Xiangxiang Wang, Yu Guo, Wencai Li, Jingliang Cheng, Xianzhi Liu, Ho-Keung Ng, Zhicheng Li, Zhenyu Zhang

**Affiliations:** ^1^Department of MRI, The First Affiliated Hospital of Zhengzhou University, Zhengzhou, China; ^2^Institute of Biomedical and Health Engineering, Shenzhen Institutes of Advanced Technology, Chinese Academy of Sciences, Shenzhen, China; ^3^Department of Pathology, The First Affiliated Hospital of Zhengzhou University, Zhengzhou, China; ^4^Department of Anatomical and Cellular Pathology, Prince of Wales Hospital, The Chinese University of Hong Kong, Hong Kong, China; ^5^Department of Neurosurgery, The First Affiliated Hospital of Zhengzhou University, Zhengzhou, China; ^6^Center for Language and Brain, Shenzhen Institute of Neuroscience, Shenzhen, China; ^7^Shenzhen Key Laboratory of Affective and Social Neuroscience, Center for Brain Disorders and Cognitive Sciences, Shenzhen University, Shenzhen, China; ^8^Department of Biomedical Sciences of Cells and Systems, University of Groningen, University Medical Center Groningen, Groningen, Netherlands

**Keywords:** medulloblastoma, radiomics, molecular subgroups, machine learning, prediction

## Abstract

The 2016 WHO classification of central nervous system tumors has included four molecular subgroups under medulloblastoma (MB) as sonic hedgehog (SHH), wingless (WNT), Grade 3, and Group 4. We aimed to develop machine learning models for predicting MB molecular subgroups based on multi-parameter magnetic resonance imaging (MRI) radiomics, tumor locations, and clinical factors. A total of 122 MB patients were enrolled retrospectively. After selecting robust, non-redundant, and relevant features from 5,529 extracted radiomics features, a random forest model was constructed based on a training cohort (*n* = 92) and evaluated on a testing cohort (*n* = 30). By combining radiographic features and clinical parameters, two combined prediction models were also built. The subgroup can be classified using an 11-feature radiomics model with a high area under the curve (AUC) of 0.8264 for WNT and modest AUCs of 0.6683, 0.6004, and 0.6979 for SHH, Group 3, and Group 4 in the testing cohort, respectively. Incorporating location and hydrocephalus into the radiomics model resulted in improved AUCs of 0.8403 and 0.8317 for WNT and SHH, respectively. After adding gender and age, the AUCs for WNT and SHH were further improved to 0.9097 and 0.8654, while the accuracies were 70 and 86.67% for Group 3 and Group 4, respectively. Prediction performance was excellent for WNT and SHH, while that for Group 3 and Group 4 needs further improvements. Machine learning algorithms offer potentials to non-invasively predict the molecular subgroups of MB.

## Introduction

Medulloblastoma (MB) is one of the most common pediatric brain tumors with high malignancy (1). In 2012, an international consensus on the molecular subgroups of MB was reached among pediatric neuro-oncology researchers (2, 3). The four putative molecular subgroups of MB were named as sonic hedgehog (SHH), wingless (WNT), Grade, 3, and Group 4, with the subgroups carrying genetic, transcriptionomic, demographic, and prognostic differences (2–6). The 2016 WHO classification of central nervous system tumors has also included the four molecular subgroups under medulloblastoma (7). The molecular subgroups now form an important factor for MB risk stratification and will be the basis for future clinical trials aimed at developing subgroup-specific treatments (3). However, the availability of molecular subgrouping of MB has been hampered by the relatively high cost of and lack of access to molecular techniques in many health settings (8).

It has been revealed that the four molecular subgroups of MB have different cellular origins (9, 10). In mice models, the WNT subgroup of MB arises from dorsal brainstem precursors, while the SHH subgroup originates from cerebellar granule neuron precursors at the upper rhombic lip (9). Therefore, tumor localization patterns may provide some clues for the molecular subgrouping of MB, and several research teams have reported promising results (11–13). Nevertheless, much more information lies in the radiographic patterns of tumor parenchyma which are not yet explored. A few studies (11, 14) have attempted to delineate different molecular subgroups of MB in terms of contrast enhancement, T2-weighted intensity, hemorrhage, necrosis, etc. However, these imaging features were human-recognized qualitative characteristics that cannot embrace all the multi-dimensional and subtle patterns presented by magnetic resonance imaging (MRI). Recently, machine learning-based radiomics analysis has been successfully applied to quantify radiographic features for identifying image biomarkers with the capability to predict genotypes and the clinical outcomes of various tumors (15, 16).

In the current study, we used a machine learning-based radiomics approach to develop a predictive model for molecular subgroups of MB (pediatric and adult) based on multi-parameter MRI, tumor location and clinical factors in a relatively large cohort (*n* = 122).

## Materials and Methods

### Patients and Molecular Subgroup Assignment

The Human Scientific Ethics Committee of The First Affiliated Hospital of Zhengzhou University has approved the protocol of this study (No. 2019-KY-176). A total of 183 patients received craniotomy for tumor resection and were pathologically diagnosed as primary MB in the Department of Neurosurgery, The First Affiliated Hospital of Zhengzhou University from January 2009 to January 2018. The 183 cases were further assessed for molecular subgroups and selected by the following criteria: (1) availability of pre-operative MRI, (2) availability of multi-parameter MRI, including axial pre-contrast T1-weighted imaging (T1), axial contrast-enhanced T1-weighted imaging (T1c), axial T2-weighted imaging (T2), axial fluid-attenuated inversion recovery (FLAIR) imaging, and apparent diffusion coefficient (ADC) maps generated from acquired diffusion-weighted imaging (DWI), and (3) availability of sufficient image quality without significant artifacts, determined by neuroradiologists (J. Yan and J. Cheng) and neurosurgeons (Z. Zhang and X. Liu). The selection procedure is depicted in Supplementary Figure 1. Clinical parameters (gender and age) were acquired from the medical record system. For molecular subgroup assessment, we used the NanoString assay for formalin-fixed paraffin-embedded tissues that were available in all the MB cases for identification of molecular subgroups (WNT, SHH, Group 3, and Group 4) as described by Northcott et al., and we used the R-script for the assay kindly provided by Dr. Michael Taylor of Sickkids, Toronto (17, 18).

### MRI Acquisition

All MR images of the enrolled patients were acquired on 3.0 T MR units (Discovery MR750, GE Healthcare, Milwaukee, WI, United States; Magnetom Trio TIM/Skyra, Siemens Healthcare, Erlangen, Germany) with 8-, 12-, or 20-channel head coil. Briefly, the brain MRI protocol included the following: (a) pre-contrast axial and sagittal T1, (b) axial T2, (c) axial FLAIR, (d) DWI, and (e) axial, sagittal, and coronal T1c acquired immediately after an intravenous administration of a 0.1-mmol/kg dose of a gadolinium-based contrast agent (gadolinium-diethylenetriamine pentaacetic acid, Bayer Healthcare, Leverkusen, Germany, or gadoteric acid meglumine salt injection, Hengrui Healthcare, Jiangsu, China) with the same parameters as the matched pre-contrast sequence. All DWI acquisitions were obtained before injection of the contrast agent and were used as a monopolar spin-echo echo-planar sequence, with diffusion sensitizing gradients encoded in the *x*, *y*, and *z* directions. ADC maps were calculated from acquired DWI with *b* = 0 and *b* = 1,000 s/mm^2^ images using the dedicated software (version 4.6, GE Healthcare, Milwaukee, WI, United States; Syngo, Siemens Healthcare, Erlangen, Germany). Details of the parameters for all the sequence acquisitions are available in Supplementary Table 1.

### Location and Hydrocephalus Status

Location features were defined as the location of the tumor geometric center and determined by a neuroradiologist (J. Yan, with 10 years of experience) according to pre-operative MRI. A second neurosurgeon (Z. Zhang, with 10 years of experience) reviewed all the location features. Any disagreement between the two raters was resolved by discussion and consensus. In this study, three locations were defined, including the midline vermian/fourth ventricle, cerebellar hemisphere, and cerebellar peduncle/cerebellopontine angle cistern (CP/CPA) (11). Moreover, hydrocephalus was defined as Evans’ index (EI) > 0.3 based on EI calculated as the ratio of the maximum width between the frontal horns of the lateral ventricles (frontal horn width) and the maximum transverse inner diameter of the skull at the same axial level (19). The EIs for all patients were determined by J. Yan and Z. Zhang.

### Image Preprocessing and Tumor Delineation

**FIGURE 1:**
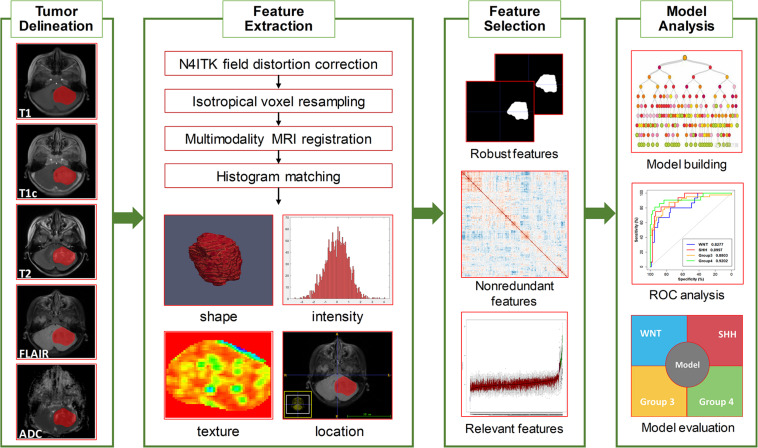
Overview of the radiomics pipeline in this study. The pipeline consisted of tumor delineation, image preprocessing, radiomics feature extraction, feature selection, model building, and model evaluation.

An overview of the radiomics analysis pipeline is shown in Figure 1. Image pre-processing was performed to normalize the intensity and the geometry. First, N4ITK algorithm was applied to correct the bias field distortion. After isotropic voxel resampling into 1 mm × 1 mm × 1 mm with linear interpolation, multi-parameter MRI rigid registration was performed with mutual information similarity metric using T1c as a template. Histogram matching was used for intensity normalization. A neuroradiologist with 10 years of experience (J. Yan), blinded to clinical, pathological, and molecular data, manually delineated the three-dimensional volume of interest (VOI) of tumor contours slice by slice using the ITK-SNAP software^1^ in the axial plane primary from FLAIR, T2, and T1c images. The VOI was defined as the region including the contrast-enhancing area, the non-enhancing area, and the necrosis area of the tumor. Specifically, the VOI contours were delineated based on FLAIR images; meanwhile, T2 and T1c were used to cross-check the tumor extension and fine-tune the tumor contour. Considering feature repeatability against intra-rater and inter-rater delineation variations, the VOI delineation process was repeated by the same neuroradiologist (J. Yan) and by another neurosurgeon (Z. Zhang) on 30 randomly selected patients, generating a test–retest data set for intra-rater repeatability analysis and a multiple delineation data set for inter-rater repeatability analysis, respectively. The segmented VOI was then overlaid with co-registered resampled T1, T1c, T2, FLAIR, and ADC images.

### Radiomics Feature Extraction

An open-source Python tool Pyradiomics was used to extract all radiomics features. Feature descriptions and mathematical definitions can be found in (20). From each VOI, high-throughput features were extracted, including (1) location features, (2) shape features, (3) intensity features, and (4) texture features. Fourteen shape features were extracted from the delineated VOI masks to describe the 3D characteristics of tumor shape. First-order intensity features were extracted to describe the intensity distributions of the voxel intensities. The texture features were extracted using five different methods, including the gray-level co-occurrence matrix, gray-level run length matrix, gray-level size zone matrix, gray-level dependence matrix, and neighborhood gray-tone difference matrix (NGTDM). The intensity features and the texture features were extracted not only from the original images but also from the transform-domain images using both wavelet transform and Laplacian of Gaussian (LoG) filter with four sigma levels (2.0, 3.0, 4.0, and 5.0). A total of 234 intensity features were extracted, where 18 were from original images, 72 were from LoG images, and 144 were from wavelet images. A total of 949 texture features were obtained, including 73 original texture features, 292 LoG texture features, and 584 wavelet features. In total, 5,929 quantitative features were extracted from five MRI sequences for each patient. The radiomics features extracted are summarized in Supplementary Table 2.

The features included three texture features and eight intensity features extracted from T1, T1c, FLAIR, or ADC images. All features were calculated from wavelet-transformed images, where H and L were high-pass and low-pass filters in wavelet transform, respectively. pFDR is short for false discovery rate-adjusted *P* value.

### Feature Selection

The features were standardized with z-score normalization to zero mean and unit variance. First, features with low repeatability against intra-rater and inter-rater delineation variations were excluded from subsequent analysis. Here the intra-rater and inter-rater repeatability for each feature was quantified by intraclass correlation coefficient (ICC) calculated between feature pairs on the intra-rater test–retest data set and inter-rater multi-delineation data set, respectively. Any feature with ICC smaller than 0.85 was discarded. To minimize feature redundancy, correlation coefficients between each pair of the remaining features were calculated. For feature pair with correlation coefficients greater than 0.90, the feature with better univariate predictive power (smaller Mann–Whitney *U* test *P* value) was retained, while the other was removed. Based on the remaining robust and non-redundant feature subset, a random forest-based wrapper feature selection algorithm Boruta (21) was used to further select the optimal all-relevant features. Boruta searched for relevant features iteratively by comparing the importance of original features with the importance of artificially added random ones and progressively removing irrelevant features. Within each iteration, a random forest algorithm was used to measure the feature importance and evaluate the model. After evaluating all possible feature combinations, the most important features for an optimal model were selected.

### Machine Learning Classification

Based on the selected feature subset, a radiomics model was built using random forest algorithm (22) for classifying the four molecular subgroups. Then, a radiomics, location, and hydrocephalus (RLH) model was built using random forest by radiomics features, tumor location, and hydrocephalus information. For comparison, a clinical model based only on clinical factors (gender and age) was also built using random forest algorithm. Furthermore, a combined radiomics, location, hydrocephalus, and clinical factors (RLHC) model was built by combining the 11 radiomics features, tumor location, hydrocephalus information, and clinical factors (gender and age), using random forest algorithm. The number of trees in a random forest algorithm was set to 500, where the Gini index was used as importance measure (22). We also evaluated three univariate parameters alone for molecular subgroup classification, including lack of contrast, location, and hemorrhage. Three prediction models using univariate logistic regression were built using each single parameter. The R packages utiml and randomForest were used for model building.

### Statistical Analysis

All statistical analyses were done with R software, version 3.6.1. A two-side *P* value of less than 0.05 was considered significant. The study population was randomly divided into a training cohort and a testing cohort at a ratio of 3:1, where the distribution of the clinical characteristics was balanced. Differences in gender, age, and molecular subgroups between the training and the testing cohorts were assessed by using Wilcoxon test or *χ*^2^ test. Differences in patient characteristics across the four molecular subgroups were assessed by using Kruskal–Wallis test. All four classification models (radiomics model, clinical model, RLH model, RLHC model, and three univariate logistic regression models) were trained on the training cohort and tested on the testing cohort. Molecular subgroup-specific classification performance (one specific class *versus* all other classes) was assessed by using receiver operating characteristic (ROC) analysis in terms of AUC, accuracy, sensitivity, and specificity. For each subgroup-specific dichotomous classification, the optimal cutoff was chosen as the maximum value of the Youden index (sensitivity + specificity − 1). All indices were calculated for both training and testing cohorts. The AUCs were statistically compared between different classifiers using DeLong analysis (23).

## Results

### Patient Characteristics

According to the selection criteria, a total of 122 patients were included in the current study. The patients were divided into a training cohort (*n* = 92) and a testing cohort (*n* = 30). Between the training and the testing cohorts, there were no significant differences in clinical characteristics [molecular subgroup (*P* = 0.8037), tumor location (*P* = 0.6365), hydrocephalus (*P* = 0.8482), gender (*P* = 0.6983), and age (*P* = 0.9028)], as shown in Table 1. Among the four molecular subgroups, significant differences in sex (*P* = 0.0004), age (*P* = 0.0001), location (*P* < 0.0001), and hydrocephalus (*P* = 0.0004) have been found, as shown in Supplementary Table 3.

**TABLE 1:** Characteristics of patients with medulloblastoma in the training cohort and testing cohort.

**Characteristic**	**Overall (*n* = 122)**	**Training cohort (*n* = 82)**	**Testing cohort (*n* = 30)**	***P*-value**
**Sex**				0.6983
Male	86 (70.5%)	64 (69.6%)	22 (73.3%)	
Female	36 (29.5%)	28 (30.4%)	8 (26.7%)	
Age (year)*	11.57 ± 10.61	11.60 ± 11.05	11.46 ± 9.12	0.9028
**Location**				0.6365
1	17 (13.9%)	13 (14.1%)	4 (13.3%)	
2	7 (5.8%)	4 (4.4%)	3 (10.0%)	
3	98 (80.3%)	75 (81.5%)	23 (76.7%)	
**Hydrocephalus**				0.8482
Absent	51 (41.8%)	38 (41.3%)	13 (43.3%)	
Present	71 (58.2%)	54 (58.7%)	17 (56.7%)	
**Molecular subgroups**				0.8037
Wingless	21 (17.2%)	15 (16.3%)	6 (20.0%)	
Sonic hedgehog	20 (16.4%)	16 (17.4%)	4 (13.3%)	
Group 3	54 (44.3%)	40 (43.5%)	14 (46.7%)	
Group 4	27 (22.1%)	21 (22.8%)	6 (20.0%)	

Data are numbers of patients, with percentages in parentheses. * Data are means ± SD. 1, cerebellar hemisphere; 2, cerebellar peduncle/cerebellopontine angle cistern; 3, midline vermian/fourth ventricle.

### Feature Selection

After the intra-rater and inter-rater robustness tests, 2,978 out of 5,929 features remained. After the redundancy reduction, 486 features were selected for subsequent analysis. The heat maps of the correlation coefficients of both the 2,978 features and the selected 486 features are shown in Supplementary Figure 2. After the Boruta feature selection, 11 most important features for an optimal model fit were finally selected, including three texture features and eight intensity features, as shown in Table 2. The result of the Boruta feature selection is shown in Supplementary Figure 3, where the boxplots of importance of all features fed to Boruta are shown. All the selected features were extracted from wavelet-transformed images. The univariate association of each selected feature with the molecular subgroup was significant (false discovery rate-adjusted *P* < 0.001).

**TABLE 2:** Eleven selected radiomics features for predicting the molecular subgroups of medulloblastoma patients.

**No.**	**Selected features**	**Type**	**Sequence**	**Transform**	**pFDR**
*f*_1_	Median	Intensity	Fluid-attenuated inversion recovery (FLAIR)	Wavelet. HHH	<0.001
*f*_2_	Gray-level co-occurrence matrix (GLCM) cluster shade	Texture	FLAIR	Wavelet. HHL	<0.001
*f*_3_	Mean	Intensity	FLAIR	Wavelet. HLL	<0.001
*f*_4_	Root mean squared	Intensity	FLAIR	Wavelet. LHL	<0.001
*f*_5_	Gray-level size zone matrix small area low gray level emphasis	Texture	T1	Wavelet. HHH	<0.001
*f*_6_	Median	Intensity	T1c	Wavelet. LHL	<0.001
*f*_7_	Skewness	Intensity	T1c	Wavelet. HLL	<0.001
*f*_8_	Maximum	Intensity	Apparent diffusion coefficient	Wavelet. LLH	<0.001
*f*_9_	GLCM inverse variance	Texture	T1	Wavelet. HLH	<0.001
*f*_10_	Skewness	Intensity	FLAIR	Wavelet. LHH	<0.001
*f*_11_	Skewness	Intensity	FLAIR	Wavelet. LHL	<0.001

### Classification Performance

**FIGURE 2:**
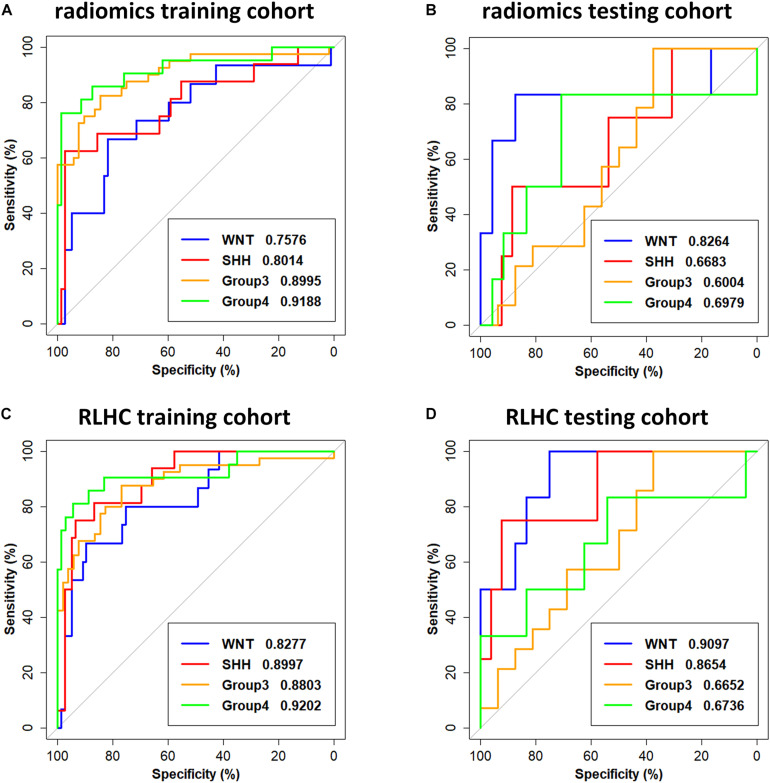
Receiver operating characteristic (ROC) curves of the radiomics model and the radiomics, location, hydrocephalus, and clinical factors (RLHC) model. **(A,B)** ROC curves of the radiomics model on the training cohort and testing cohort, respectively. **(C,D)** ROC curves of the RLHC model on the training cohort and testing cohort, respectively.

The subgroup-specific ROC curves for both training and testing cohorts of the radiomics model and the RLHC model are shown in Figure 2. The ROC curves of the clinical model and the RLH model are shown in Supplementary Figure 4. The AUCs of the 11-feature radiomics model were 0.8264 for WNT, 0.6683 for SHH, 0.6004 for Group 3, and 0.6979 for Group 4 in the testing cohort. When combining the 11 imaging features with tumor location and hydrocephalus information, the AUCs of the RLH model were 0.8403 for WNT, 0.8317 for SHH, 0.6451 for Group 3, and 0.6111 for Group 4 in the testing cohort. In the training cohort, significant differences of AUCs between the radiomics model and the RHL model were found for both WNT and SHH subgroups (DeLong *P* < 0.01). However, in the testing cohort, no significant AUC difference between the radiomics model and the RLH model was found for any subgroup.

The AUCs of the clinical model in the testing cohort were 0.8681 for WNT, 0.7163 for SHH, 0.5469 for Group 3, and 0.5035 for Group 4. After incorporating the clinical information into the RLH model, the AUCs of the RLHC model in the testing cohort further improved to 0.9097 for WNT, 0.8654 for SHH, 0.6652 for Group 3, and 0.6736 for Group 4. In addition, among the three univariate models, the location-based model achieved AUCs of 0.5352 for WNT, 0.8410 for SHH, 0.6610 for Group 3, and 0.5311 for Group 4. The contrast- and hemorrhage-based models failed in subgroup prediction (AUCs: 0.5000 to 0.5127). The subgroup-specific ROC curves of the location model are shown in Supplementary Figure 5. In the training cohort, significant differences of AUCs between the radiomics and the RLHC models were found for both WNT and SHH subgroups (DeLong *P* < 0.01). In the testing cohort, a significant AUC difference between the RLHC model and the radiomics model was found only for the SHH subgroup (DeLong *P* = 0.04). The classification performance of the radiomics model and that of the RLHC model in both training and testing cohorts are summarized in Tables 3, 4, respectively. The performance of the clinical model and that of the RLH model are shown in Supplementary Tables 4, 5, respectively.

**TABLE 3:** Summary of the subgroup-specific classification performance of the radiomics model.

**Molecular subgroups**	**Cohorts**	**Area under the curve**	**ACC (%)**	**SEN (%)**	**SPE (%)**
Wingless	Training	0.7576 (0.6113–0.9039)	79.35 (52.15–90.22)	66.67 (46.67–100.00)	81.82 (44.16–97.40)
	Testing	0.8264 (0.5597–1)	86.67 (76.67–100.00)	50.00 (50.00–100.00)	95.83 (75.00–100.00)
Sonic hedgehog	Training	0.8014 (0.6598–0.943)	91.30 (65.22–95.65)	62.50 (43.75–93.75)	97.37 (59.21–100.00)
	Testing	0.6683 (0.361–0.9755)	83.33 (33.33–93.33)	75.00 (50.00–100.00)	80.77 (23.08–100.00)
Group 3	Training	0.8995 (0.8325–0.9665)	83.70 (78.26–91.30)	82.50 (62.50–95.06)	84.62 (69.23–100.00)
	Testing	0.6004 (0.3887–0.8122)	60.00 (56.67–80.00)	78.57 (72.86–100.00)	43.75 (18.75–93.75)
Group 4	Training	0.9188 (0.8368–1)	93.48 (79.35–97.83)	76.19 (66.67–100.00)	98.59 (77.46–100.00)
	Testing	0.6979 (0.4042–0.9916)	76.67 (60.00–90.00)	83.33 (50.00–100.00)	75.00 (54.17–95.83)

*ACC, SEN, and SPE are short for accuracy, sensitivity, and specificity, respectively. The 95% confidence interval for each index is shown.*

**TABLE 4:** Summary of the subgroup-specific classification performance of the RLHC model.

**Molecular subgroups**	**Cohorts**	**Area under the curve**	**ACC (%)**	**SEN (%)**	**SPE (%)**
Wingless	Training	0.8277 (0.7133–0.9421)	85.87 (53.26–93.48)	66.67 (53.33–100.00)	89.61 (44.16–97.40)
	Testing	0.9097 (0.7987–1.0000)	80.00 (70.00–96.67)	83.33 (83.33–100.00)	79.17 (62.50–100.00)
Sonic hedgehog	Training	0.8997 (0.8236–0.9758)	90.22 (65.22–95.65)	75.00 (68.75–100.00)	93.42 (57.89–98.68)
	Testing	0.8654 (0.6609–1)	86.67 (56.67–100.00)	75.00 (75.00–100.00)	88.46 (50.00–100.00)
Group 3	Training	0.8803 (0.8059–0.9547)	81.52 (76.09–90.22)	87.50 (65.00–97.50)	76.92 (67.31–98.08)
	Testing	0.6652 (0.4667–0.8636)	70.00 (60.00–83.33)	100.00 (35.71–100.00)	50.00 (25.00–100.00)
Group 4	Training	0.9202 (0.8372–1.00)	92.39 (82.61–96.74)	76.19 (71.43–100.00)	97.18 (80.28–100.00)
	Testing	0.6736 (0.3714–0.9759)	86.67 (50.00–93.33)	33.33 (16.67–100.00)	100.00 (41.67–100.00)

*RLHC model means the model combining the 11 selected radiomics features, the tumor location, the hydrocephalus information, and the clinical factors. ACC, SEN, and SPE are short for accuracy, sensitivity and specificity, respectively. The 95% confidence interval for each index is shown.*

**FIGURE 3:**
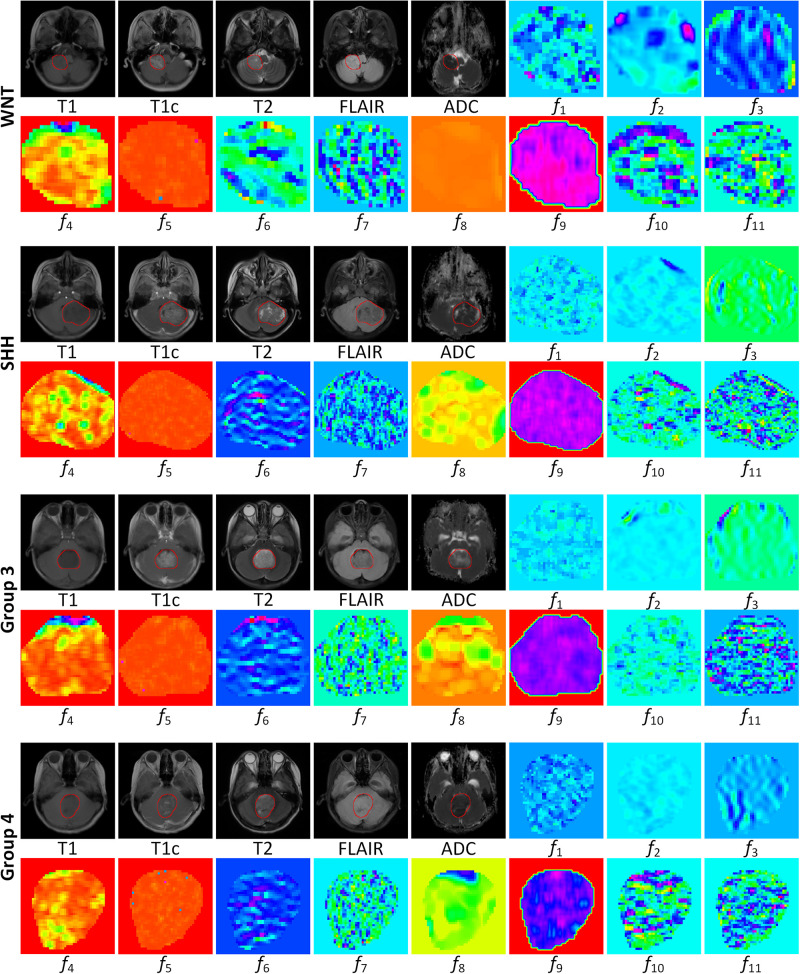
MR images and corresponding feature maps of the selected 11 features for a wingless patient, a sonic hedgehog patient, a group 3 patient, and a group 4 patient. The delineated tumor contour was overlapped on the MR images. The radiomics features *f*_1_–*f*_11_ are defined in Table 2. The feature maps visualized the intratumoral variations of the image patterns, revealing the association of the radiomics features with the molecular subgroups.

**FIGURE 4:**
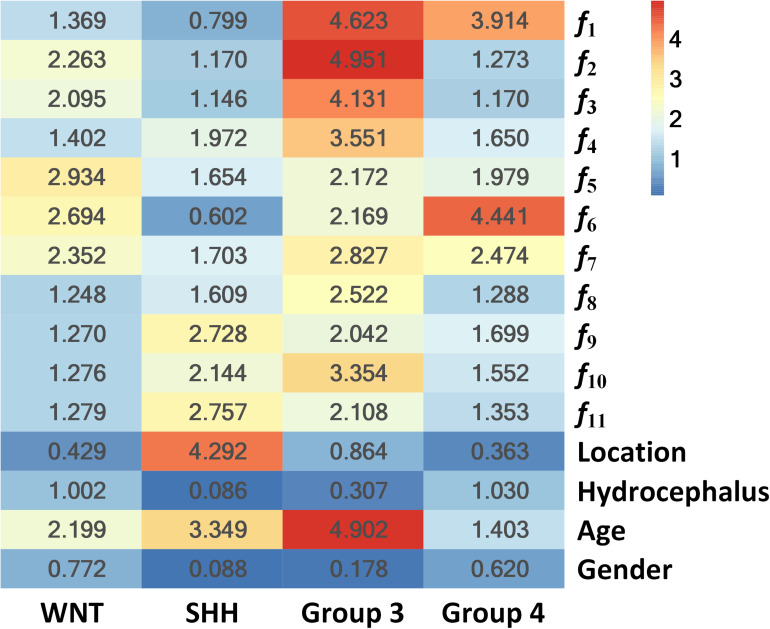
Heat map of the subgroup-specific importance of all parameters used in subgroup classification.

To further illustrate the relevance of the selected 11 radiomics features with the four molecular subgroups, typical MR images and corresponding feature maps are presented in Figure 3 for a WNT patient, a SHH patient, a Group 3 patient, and a Group 4 patient, respectively. To describe the univariate contribution of each parameter used (the selected 11 radiomics features, tumor location, hydrocephalus information, age, and gender) to subgroup classification, a heat map of the subgroup-specific parameter importance in classification is shown in Figure 4. The meanings of the 11 radiomics features are detailed in Supplementary Table 6.

The importance values are calculated as the Gini index in building the RLHC model, indicating the univariate contribution to the classification. A larger value means more importance in classifying a specific subgroup. For example, the feature *f*_6_ was the most important in Group 4 classification, while tumor location contributed most in SHH classification.

## Discussion

In this study, data from clinical factors (age and gender) and radiographic information (tumor location, hydrocephalus, and radiomic features from tumor parenchyma) were utilized to develop predictive models for the molecular subgroups of MB. Compared to other reports focusing on the relationship of MRI features and the molecular subgroups of MB (8, 11–14, 24), the current study has several strengths. First, this study used a machine learning method to analyze nearly all the suitable information extracted from routine pre-operative examinations for MB patients. Third, T1, T1c, T2, FLAIR, and ADC MR sequences were used to provide radiomic signatures, which were also the most integrated MR sequences to date.

The consensus of the four molecular subgroups is now the basis for MB patient stratification and design of many clinical trials (25). Nonetheless, the assignment of molecular subgroups of MB in routine clinical situation is still a challenge for many institutions with limited resources (8, 17). The NanoString assay demonstrated by Northcott et al. is an accurate, reproducible, and rapid method for the molecular subgrouping of MB (17), but this method is only recently available for research use in a few medical centers in China, India, and Brazil (26–28). Therefore, it is of great clinical and social significance to be able to predict the molecular subgroups of MB with the information provided in routine examinations conducted in daily medical practice. Another merit of the non-invasive assignment of molecular subgroup before surgical intervention is its guidance for neurosurgical strategies. EM Thompson et al. revealed that no significant survival benefit existed for greater extent of resection for patients with WNT, SHH, and Group 3 tumors and suggested that the surgical removal of small residual of MB tumors should not be pursued if there is clinical risk of neurological sequelae, especially in these three molecular subgroups (6).

It is conceivable that the optimal prediction model for the molecular subgroups of MB in clinical situation should include data from pre-operative routine examinations as much as possible. There are two aspects of data suitable for developing the prediction model, namely, clinical and radiographic parameters. Clinical parameters (age and gender) have been demonstrated to be associated with the molecular subgroups of MB (2, 4, 5). The bulk of information lies in the radiographic features presented in MRI, and recent progress in radiomic algorithms has enabled researchers to extract high-dimensional radiographic patterns indiscernible to the human eye and analyze them quantitatively instead of qualitatively (15, 16, 29). Radiomics has been extensively investigated in several major cancers, such as lung cancer, breast cancer, hepatocellular carcinoma, and gliomas (16, 30). To date, a limited number of studies have explored the relationship between MRI features and the molecular subgroups of MB (8, 11–14, 24), most of which characterized the qualitative imaging features, such as location, hydrocephalus, enhancement patterns, T2-weighted characteristics, hemorrhage, necrosis, and calcification. Prior studies have shown that location is a key feature predictive of molecular subgroups. SHH tumors occur most frequently in the cerebellar hemisphere, while Group 3 and Group 4 tumors often arise in the midline vermian/fourth ventricle, and most WNT tumors involve both the midline and the CP/CPA regions (11–14, 24, 31). Moreover, the enhancement patterns differ across MB subgroups. For instance, WNT tumors lie at one end of the spectrum, with homogeneous enhancement involving almost the entire tumor, while Group 4 tumors lie at the other end, with a large proportion of non-enhancing or very faintly enhancing tumor (11, 13, 14). In addition, Dasgupta et al. have reported that hydrocephalus may have an important role in discriminating between subgroups (14).

Recently, M Iv et al. extracted T2 and T1c radiomic features from pediatric MB to develop a predictive model for molecular subgrouping (8). Their models reached acceptable performance for predicting SHH and Group 4 subgroups with AUCs of 0.70–0.73 and 0.76–0.80, respectively, while the AUCs for predicting WNT and Group 3 reduced to 0.45–0.72 and 0.39–0.57, respectively. The reasons for the latter may be related to the limited MR sequences, the relatively small sample size, the lack of information of tumor location, and clinical parameters. Previous imaging–genomics studies in patients with gliomas have shown that extracting radiomics features from multiple MR sequences significantly benefited the prediction performance (32–34). In this study, we extracted radiomics features from five conventional MRI sequences (T1, T1c, T2, FLAIR, and ADC) and finally constructed an 11-feature-based model to predict the molecular subgroups of MB. The visualized feature maps of four patients in Figure 3 give an illustrative example of how the selected 11 radiomics features were associated with the subgroups.

Furthermore, since tumor location and hydrocephalus status were revealed to be significantly related to the molecular subgroups of MB (11–14, 24, 31), these two aspects were evaluated and added to the predictive model. The resulting RLH model achieved improved AUCs of 0.8403 and 0.8317 for predicting WNT and SHH, respectively, as shown in Supplementary Table 5. The heat map of parameter importance in Figure 4 shows the location which contributed the most in predicting the SHH subgroup. Moreover, by combining the 11 radiomics features, location, hydrocephalus, gender, and age, the resulting RLHC model predicted WNT and SHH with further improved AUCs of 0.9097 and 0.8654, respectively, while the overall accuracy for predicting Group 3 and Group 4 was improved to 70 and 86.67%, respectively, as shown in Table 4. Our predictive model outperformed current qualitative criteria in predicting a WNT medulloblastoma but had poorer performance in predicting Group 3 and Group 4 medulloblastoma (14). This may be due to the specific radiomics features used in our study which better reflect the underlying biological processes or cellular functions associated with the WNT subtype. This warrants further investigation on a relatively larger study cohort with paired MRI and RNA sequencing data. Recently, one study revealed that Group 3 and Group 4 MBs both exhibited a developmental trajectory from primitive progenitor-like to more mature neuronal-like cells (10). The heat map in Figure 4 also shows that age was of great importance in the prediction of SHH and Group 3 subgroups.

There are several limitations concerning the current study. First, this study utilized NanoString assay for molecular subgrouping, which is not a calibrated assay. Second, the sample size of the current cohort is still insufficient to utilize the full potential of radiomics features especially when machine learning approach was applied. A prospective, multicenter collaborative study with much greater number of participants will further improve the performance and the generalization of the predictive model. Third, there are several advanced MRI sequences, such as magnetic resonance spectroscopy (MRS) and dynamic susceptibility contrast perfusion MR, that were not included since these were unavailable in most of the cohorts. It has been reported that a subgroup classifier based on MRS was able to discriminate between SHH subgroup and Group 3 and Group 4 subgroups with satisfying accuracy (35). These clinically used MRI sequences are recommended to be included in the design of a future study. Finally, the underlying mechanisms why radiomics features could reflect the molecular subgroups of MB remain elusive and need extensive investigations. Analyzing data of high-throughput sequencing of tumor specimens and paired radiographic features by advanced artificial intelligence algorithms may be the way to gain insight to these mechanisms.

## Conclusion

In summary, by using a machine learning algorithm, clinical and radiographic information from pre-operative routine examinations were demonstrated to be capable of predicting the molecular subgroups of MB with high accuracy. The prediction performance of the model for WNT (AUC 0.9097 and accuracy 80%) and SHH (AUC 0.8654 and accuracy 86.67%) subgroups was excellent in the testing cohort, while that for Group 3 and Group 4 MB needs further improvements. Machine learning algorithms using data from routine examinations hold great promises for non-invasive pre-operative prediction of the molecular subgroups of MB.

## Data Availability Statement

The raw data supporting the conclusions of this article will be made available by the authors, without undue reservation.

## Ethics Statement

The studies involving human participants were reviewed and approved by the Human Scientific Ethics Committee of The First Affiliated Hospital of Zhengzhou University (No. 2019-KY-176). Written informed consent to participate in this study was provided by the participants’ legal guardian/next of kin. Written informed consent was obtained from the individual(s), and minor(s)’ legal guardian/next of kin, for the publication of any potentially identifiable images or data included in this article.

## Author Contributions

ZZ, JY, ZiL, H-KN, WiW, XL, KK-WL, BY, HG, WL, and JC contributed to research conception, design, and manuscript revision. ZZ, JY, ZiL, LL, and YaZ contributed to data processing, statistical analysis, and drafting of the manuscript. KK-WL, H-KN, WiW, and LW contributed to the detection of molecular subgroups of medulloblastoma. JY and ZZ took charge in delineating the volume of interest of the tumor contours and in defining the locations and hydrocephalus of the tumors. JY, KL, and XW downloaded the MRI data from the picture archiving and communication system and sorted them. KL, YnZ, WD, DP, HZ, TS, CS, WnW, ZeL, XH, XW, and YG took charge in the acquisition of tissue specimens and clinical data. All authors contributed to the article and approved the submitted version.

## Conflict of Interest

The authors declare that the research was conducted in the absence of any commercial or financial relationships that could be construed as a potential conflict of interest.

## Footnotes

1 http://www.itksnap.org

## Abbreviations

ADC: apparent diffusion coefficient
AUC: area under ROC curve
CH: cerebellar hemisphere
CNS: central nervous system
CP: cerebellar peduncle
CPA: cerebellopontine angle cistern
DWI: diffusion-weighted imaging
EI: Evans’ index
FFPE: formalin-fixed paraffin-embedded
FLAIR: fluid-attenuated inversion recovery
GLCM: gray-level co-occurrence matrix
GLDM: gray level dependence matrix
GLRLM: gray-level run length matrix
GLSZM: gray level size zone matrix
ICC: intraclass correlation coefficient
LoG: Laplacian of Gaussian
MB: medulloblastoma
MRI: magnetic resonance imaging
NGTDM: neighborhood gray-tone difference matrix
RLH: radiomics, location and hydrocephalus
RLHC: radiomics, location, hydrocephalus and clinical factors
ROC: receiver operating characteristic
SHH: sonic hedgehog subgroup
T1: precontrast T1-weighted imaging
T1c: contrast-enhanced T1-weighted imaging
T2: T2-weighted imaging
VOI: volume of interest
WNT: wingless subgroup.
