# Supplementary material for: Radiomic Features From Multi-Parameter MRI Combined With Clinical Parameters Predict Molecular Subgroups in Patients With Medulloblastoma
Source: Front Oncol. 2020 Oct 2;10:558162. doi: 10.3389/fonc.2020.558162 (PMC7566191; doi:10.3389/fonc.2020.558162)
Supplement: Supplementary file 1 [file Data_Sheet_1.docx]

**Figure S1** The patient selection procedure in this study.


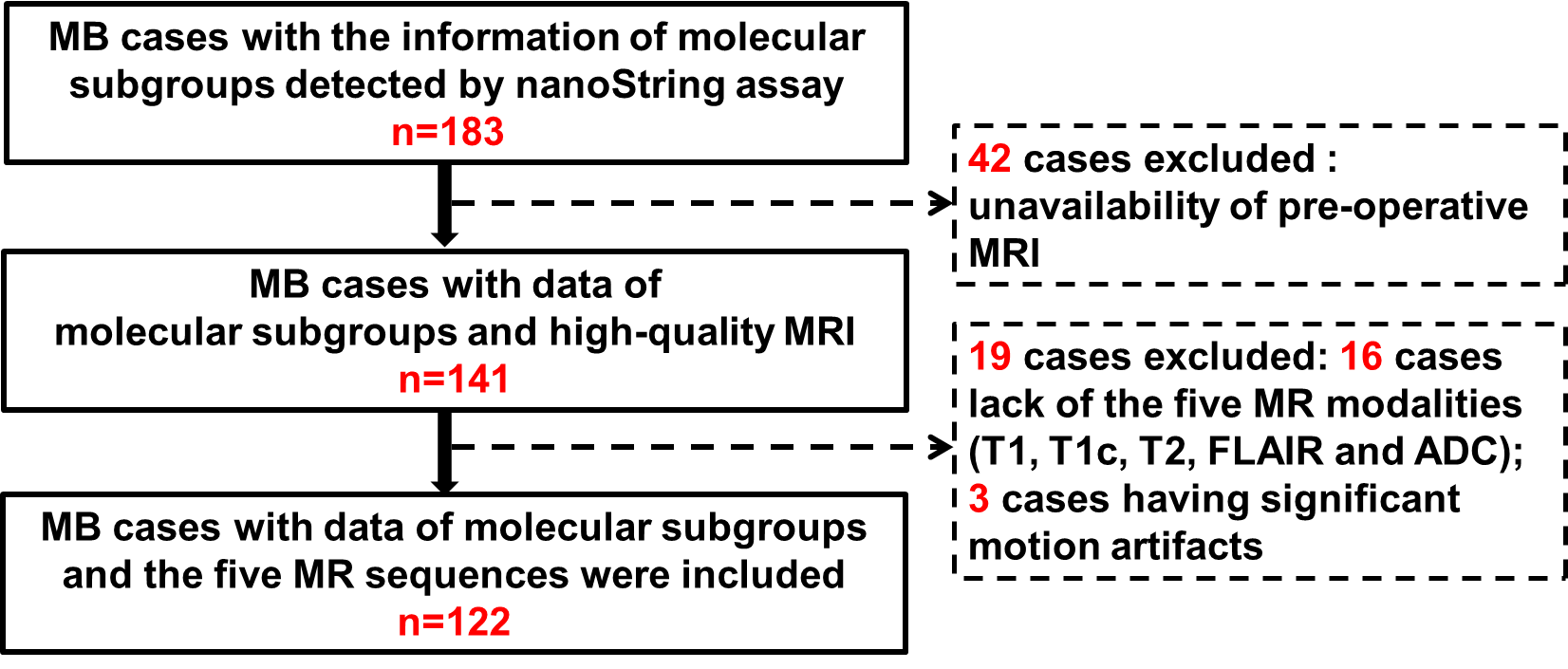


**Figure S2** The heatmaps of the correlation coefficients of the imaging features.

(a) The correlation coefficient heatmap of the 2978 features before redundancy removal. (b) The correlation coefficient heatmap of the selected 486 nonredundant features. The correlation coefficients were normalized to their z-scores (zero mean and unit variance) and then were clustered. The brighter the red (blue) color, the higher (lower) the correlation of a feature pair. The heatmaps in (a) and (b) showed that the correlations between feature pairs were reduced after applying the redundancy removal.


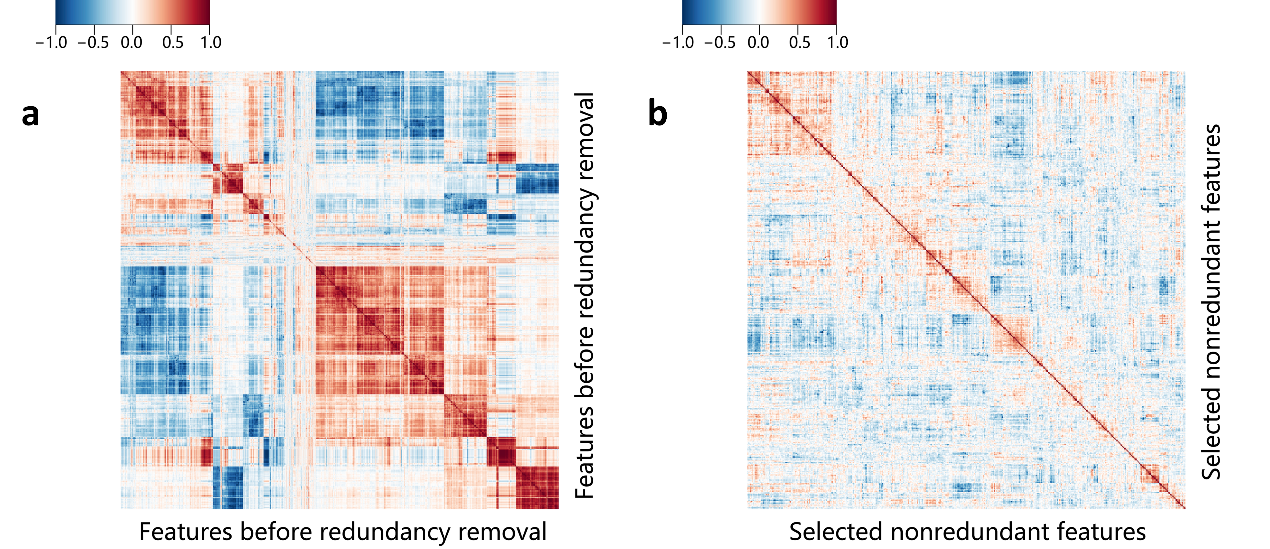


**Figure S3** The result of the Boruta feature selection.

The y-axis represents the box plots of the feature importance generated by the Boruta algorithm, while the x-axis represents all 486 features fed to the Boruta algorithm. Finally, 11 features with largest importance values were selected, as shown in green. The discarded features with smaller importance values were shown in red.


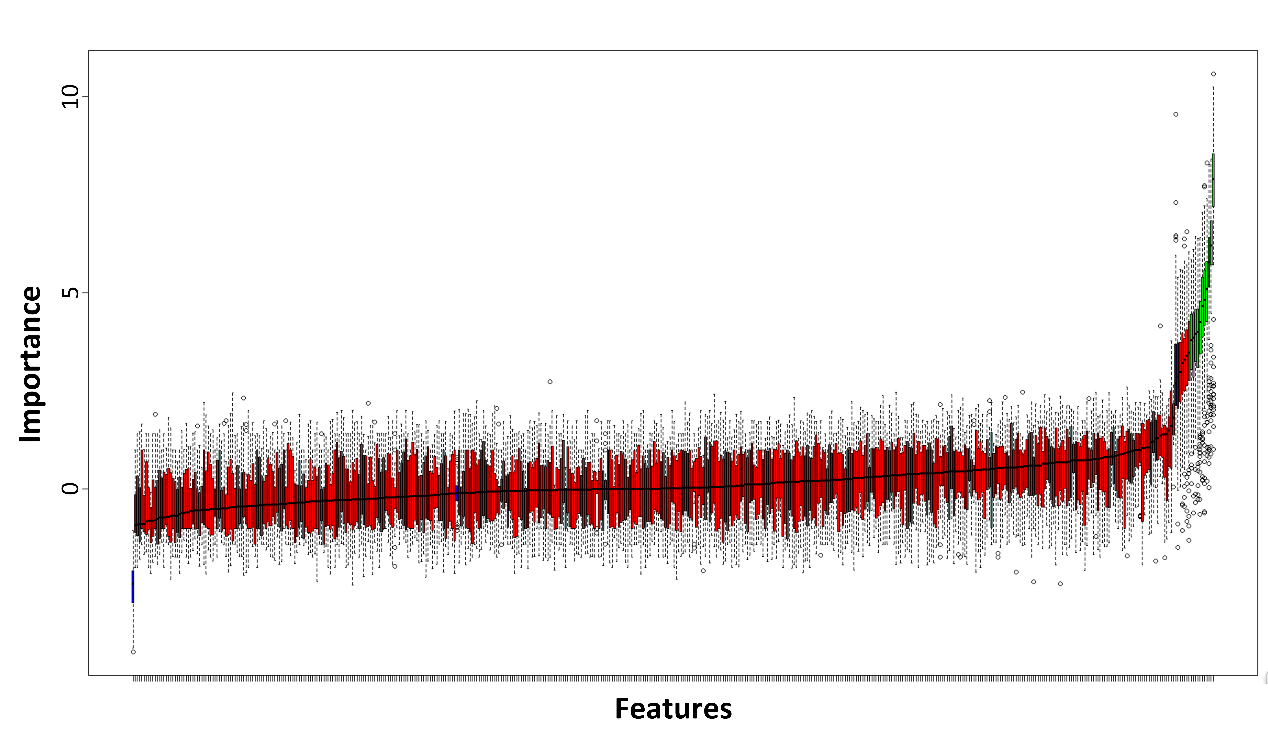


**Figure S4** The ROC curves of the clinical model and the RLH (radiomics, location, and hydrocephalus) model.

(a) and (b): ROC curves of the clinical model on the training cohort and testing cohort, respectively. (c) and (d): ROC curves of the RLH model on the training cohort and testing cohort, respectively.


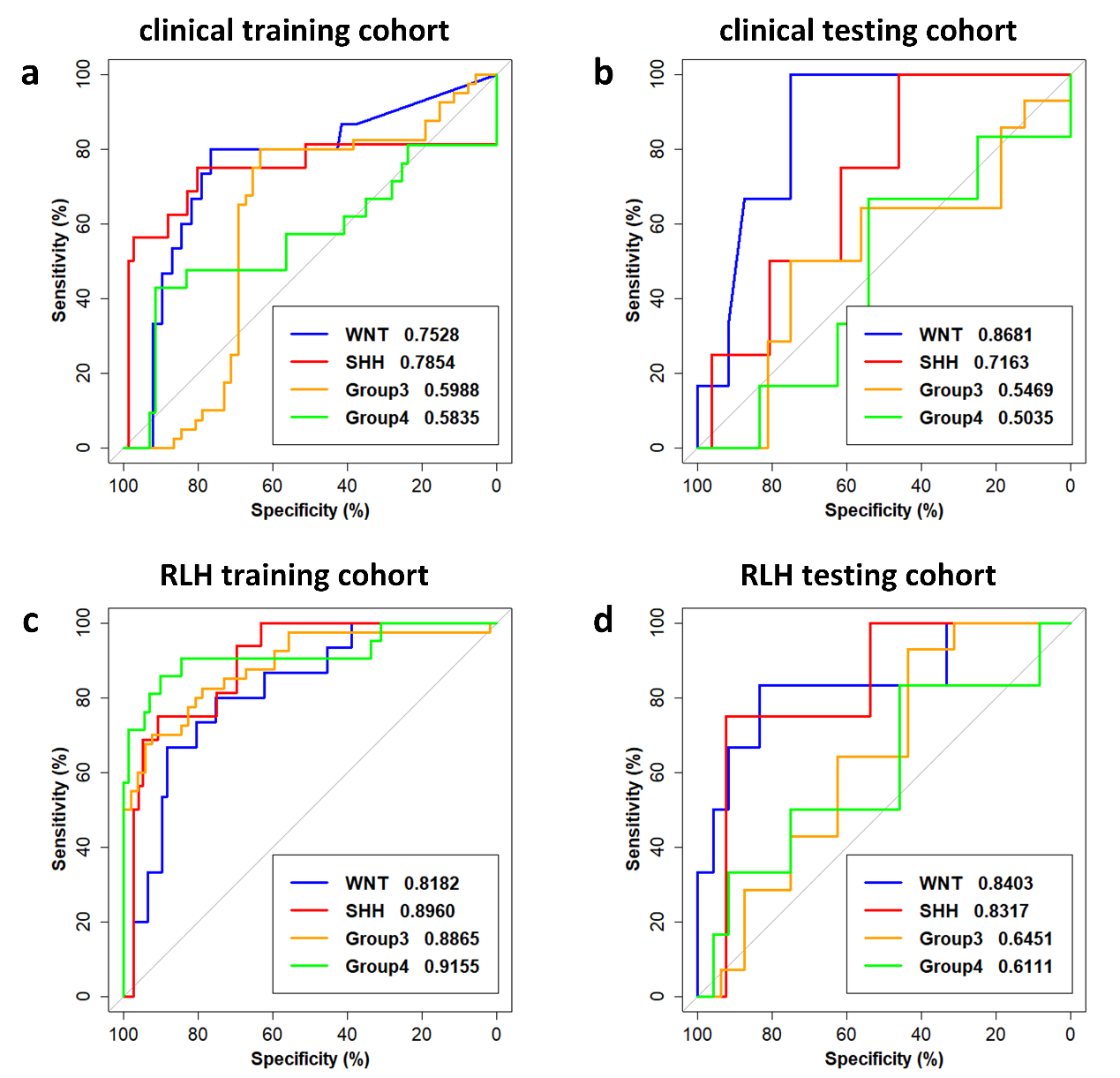


**Figure S5** The ROC curves of the of the location-based univariate logistic model.

(a) and (b): ROC curves of the location model on the training cohort and testing cohort, respectively.


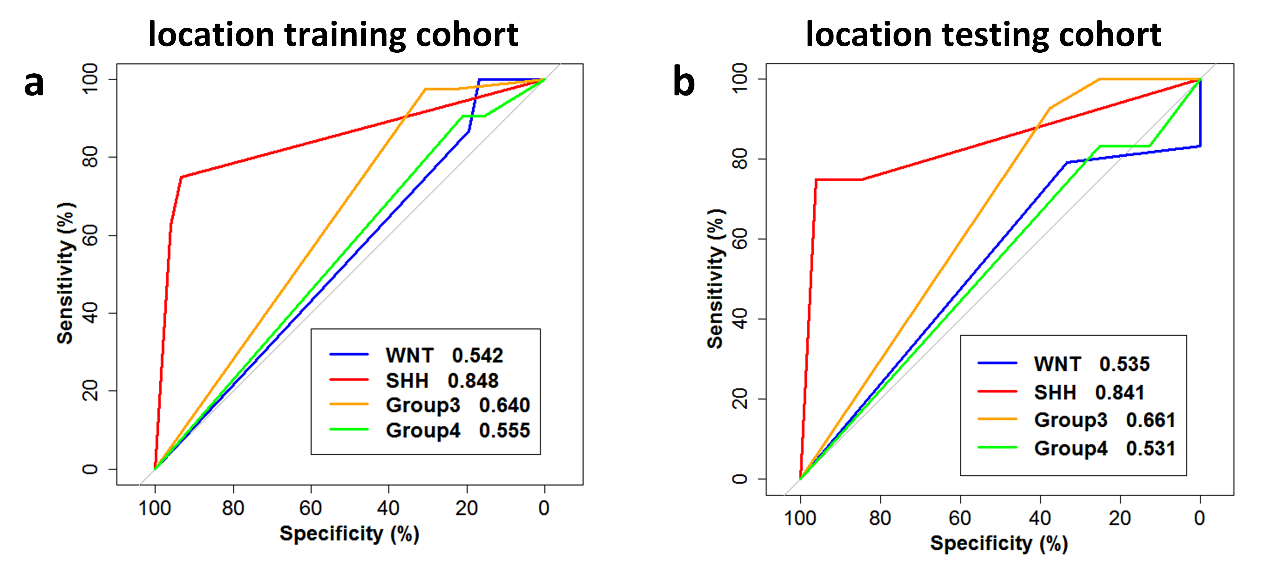


**Table S1** MR sequence parameters used in this study.

|  | **Siemens Skyra** | **Siemens Trio TIM** | **GE Discovery MR750** |
| --- | --- | --- | --- |
| Field strength (T) | 3 | 3 | 3 |
| Head coil channel | 20 | 12 | 8 |
| T1/T1c |  |  |  |
| TR (ms) | 220 | 260 | 1750 |
| TE (ms) | 3 | 3 | 24 |
| Section thickness (mm) | 5 | 5 | 5 |
| Intersection gap(mm) | 1.5 | 1.5 | 1.5 |
| FA (°) | 70 | 70 | 111 |
| NEX | 1 | 1 | 1 |
| FOV (mm) | 240×240 | 240×240 | 240×240 |
| Matrix | 288×216 | 256×162 | 320×256 |
| T2 |  |  |  |
| TR (ms) | 5000 | 3800 | 5390 |
| TE (ms) | 117 | 89 | 79 |
| Section thickness (mm) | 5 | 5 | 5 |
| Intersection gap (mm) | 1.5 | 1.5 | 1.5 |
| FA (°) | 90 | 120 | 142 |
| NEX | 1 | 1 | 1 |
| FOV (mm) | 240×240 | 240×240 | 240×240 |
| Matrix | 320×320 | 320×211 | 512×512 |
| FLAIR |  |  |  |
| TR (ms) | 6500 | 4500 | 8400 |
| TE (ms) | 85 | 93 | 150 |
| TI (ms) | 2127 | 1670 | 2100 |
| Section thickness (mm) | 5 | 5 | 5 |
| Intersection gap (mm) | 1.5 | 1.5 | 1.5 |
| FA (°) | 150 | 130 | 111 |
| NEX | 1 | 1 | 1 |
| FOV (mm) | 240×240 | 240×240 | 240×240 |
| Matrix | 256×179 | 256×194 | 256×256 |
| DWI |  |  |  |
| TR (ms) | 4600 | 3500 | 6000 |
| TE (ms) | 80 | 85 | 87 |
| Section thickness (mm) | 5 | 5 | 5 |
| Intersection gap (mm) | 1.5 | 1.5 | 1.5 |
| B value (s/mm^2^) | 0, 1000 | 0, 1000 | 0, 1000 |
| FA (°) | 90 | 90 | 90 |
| NEX | 1 | 1 | 1 |
| FOV (mm) | 240×240 | 240×240 | 240×240 |
| Matrix | 192×192 | 152×152 | 160×160 |

**Table S2** A summary of radiomics features extracted in this study.

| **Feature Subgroup** | **Feature Name** |
| --- | --- |
| Shape Features | Elongation, Flatness, Least Axis Length, Major Axis Length, Maximum 2D Diameter Column, Maximum 2D Diameter Row, Maximum 2D Diameter Slice, Maximum 3D Diameter, Mesh Volume, Minor Axis Length, Sphericity, Surface Area, Surface Volume Ratio, Voxel Volume |
| Intensity Features | Maximum, Median, Minimum, Mean, Energy, Entropy, Variance, Kurtosis, Root Mean Square, Skewness, 10th Percentile, 90th Percentile, Mean Absolute Deviation, Uniformity, Range, Robust Mean Absolute Deviation, Total Energy, Interquartile Range |
| GLCM Features | Contrast, Correlation, Autocorrelation, Cluster Tendency, Sum Average, Sum Entropy, Sum Squares, Difference Average, Difference Variance, Difference Entropy, Cluster Prominence, Cluster Shade, Maximum Probability, Inverse Difference Moment, Informational Measure of Correlation 1/2, Inverse Difference Moment Normalized, Inverse Difference Normalized, Inverse Difference, Inverse Variance, Maximal Correlation Coefficient, Joint Average, Joint Energy, Joint Entropy |
| GLDM Features | Dependence Entropy, Dependence Non-Uniformity, Dependence Non-Uniformity Normalized, Dependence Variance, Gray-Level Non-Uniformity, Gray-Level Variance, High Gray-Level Emphasis, Large Dependence Emphasis, Large Dependence High Gray-Level Emphasis, Large Dependence Low Gray-Level Emphasis, Low Gray-Level Emphasis, Small Dependence Emphasis, Small Dependence High Gray-Level Emphasis, Small Dependence Low Gray-Level Emphasis |
| GLRLM Features | Gray-Level Non-uniformity, Gray-Level Non-uniformity Normalized, Gray-Level Variance, High Gray-Level Run Emphasis, Long Run Emphasis, Long Run High Gray-Level Emphasis, Long Run Low Gray-Level Emphasis, Low Gray-Level Run Emphasis, Run Entropy, Run Length Non-Uniformity, Run Length Non-Uniformity Normalized, Run Percentage, Run Variance, Short Run Emphasis, Short Run High Gray-Level Emphasis, Short Run Low Gray-Level Emphasis |
| GLSZM Features | Gray-Level Non-Uniformity, Gray-Level Non-Uniformity Normalized, Gray-Level Non-Uniformity Normalized, High Gray-Level Zone Emphasis, Large Area Emphasis, Large Area High Gray-Level Emphasis, Large Area Low Gray-Level Emphasis, Low Gray-Level Zone Emphasis, Size Zone Non-Uniformity, Size Zone Non-Uniformity Normalized, Small Area Emphasis, Small Area High Gray-Level Emphasis, Small Area Low Gray-Level Emphasis, Zone Entropy, Zone Percentage, Zone Variance |
| NGTDM Features | Coarseness, Contrast, Busyness, Complexity, Strength |

**Table S3** Distribution of patient characteristics across the four molecular subgroups.

| **Characteristic** | **WNT**  **(n=21)** | **SHH**  **(n=20)** | **Group 3**  **(n=54)** | **Group 4**  **(n=27)** | ***P* Value** |
| --- | --- | --- | --- | --- | --- |
| Sex |  |  |  |  | 0.6983 |
| Male | 86 (70.5%) | 64 (69.6%) | 22 (73.3%) |  |  |
| Female | 36 (29.5%) | 28 (30.4%) | 8 (26.7%) |  |  |
| Age(year)* | 11.57±10.61 | 11.60±11.05 | 11.46±9.12 |  | 0.9028 |
| Location**^†^** |  |  |  |  | 0.6365 |
| 1 | 17 (13.9%) | 13 (14.1%) | 4 (13.3%) |  |  |
| 2 | 7 (5.8%) | 4 (4.4%) | 3 (10.0%) |  |  |
| 3 | 98 (80.3%) | 75 (81.5%) | 23 (76.7%) |  |  |
| Hydrocephalus |  |  |  |  | 0.8482 |
| Absent | 51 (41.8%) | 38 (41.3%) | 13 (43.3%) |  |  |
| Present | 27 (22.1%) | 21 (22.8%) | 6 (20.0%) |  |  |

Data are numbers of patients, with percentages in parentheses.

*Data are means ± standard deviations.

†Location: 1: cerebellar hemisphere (CH), 2: cerebellar peduncle/cerebellopontine angle cistern (CP/CPA), 3: the midline vermian/fourth ventricle.

**Table S4** A summary of the subgroup-specific classification performance of the clinical model. ACC, SEN and SPE were short for accuracy, sensitivity and specificity, respectively. The 95% confidence interval for each index was shown.

| **Molecular subgroups** | **Cohorts** | **AUC** | **ACC (%)** | **SEN (%)** | **SPE (%)** |
| --- | --- | --- | --- | --- | --- |
| WNT | Training | 0.7528  (0.6082-0.8974) | 77.17  (69.57-88.04) | 80.00  (60.00-100.00) | 76.62  (68.80-89.61) |
|  | Testing | 0.8681  (0.7372-0.9989) | 83.33  (66.67-96.67) | 100.00  (83.33-100.00) | 79.17  (58.33-95.83) |
| SHH | Training | 0.7854  (0.6323-0.9384) | 90.22  (75.00-94.57) | 56.25  (43.75-93.75) | 97.37  (73.68-100.00) |
|  | Testing | 0.7163  (0.4716-0.9611) | 63.33  (40.00-93.33) | 100.00  (50.00-100.00) | 57.69  (30.77-100.00) |
| Group 3 | Training | 0.5988  (0.4754-0.7345) | 70.65  (61.96-79.35) | 80.00  (65.00-92.50) | 65.38  (51.92-76.92) |
|  | Testing | 0.5469  (0.3296-0.7642) | 66.67  (53.33-80.00) | 64.29  (28.57-100.00) | 75.00  (18.75-93.75) |
| Group 4 | Training | 0.5835  (0.4269-0.7401) | 80.43  (70.65-86.96) | 42.86  (23.81-71.43) | 91.55  (77.46-97.18) |
|  | Testing | 0.5035  (0.2646-0.7632) | 56.67  (33.33-80.00) | 83.33  (16.67-100.00) | 54.17  (20.83-91.67) |

**Table S5** A summary of the subgroup-specific classification performance of the RLH (radiomics, location, and hydrocephalus) model.

RLH model means the model combining the 11 selected radiomics features, the tumor location, and the hydrocephalus information. ACC, SEN and SPE were short for accuracy, sensitivity and specificity, respectively. The 95% confidence interval for each index was shown.

| **Molecular subgroups** | **Cohorts** | **AUC** | **ACC (%)** | **SEN (%)** | **SPE (%)** |
| --- | --- | --- | --- | --- | --- |
| WNT | Training | 0.8182  (0.7093-0.927) | 84.78  (53.26-91.30) | 66.67  (60.00-100.00) | 88.31  (44.16-93.51) |
|  | Testing | 0.8403  (0.6248-1.0000) | 86.67  (66.67-96.67) | 83.33  (50.00-100.00) | 87.50  (58.33-100.00) |
| SHH | Training | 0.8960  (0.8218-0.9701) | 90.22  (66.30-95.65) | 68.75  (68.75-100.00) | 94.74  (59.21-98.68) |
|  | Testing | 0.8317  (0.6159-1.0000) | 90.00  (53.33-100.00) | 75.00  (50.00-100.00) | 92.31  (46.15-100.00) |
| Group 3 | Training | 0.8865  (0.8161-0.9570) | 80.43  (75.00-90.22) | 82.50  (60.00-97.50) | 78.85  (61.54-100.00) |
|  | Testing | 0.6451  (0.4411-0.849) | 63.33  (56.67-83.33) | 92.86  (42.86-100.00) | 37.50  (18.75-93.75) |
| Group 4 | Training | 0.9155  (0.8275-1.0000) | 91.30  (82.61-96.74) | 90.48  (71.43-100.00) | 91.55  (80.28-100.00) |
|  | Testing | 0.6111  (0.325-0.8972) | 80.00  (30.00-90.00) | 33.33  (16.67-100.00) | 91.67  (12.50-100.00) |

**Table S6** The meanings of the 11 radiomics features constituting the radiomics model.

To fully characterize the image phenotypes within the tumor, we extracted radiomics features from not only the original medical images but also the transformed, or derived images by using wavelet or Laplacian of Gaussian (LoG) filters onto the original images. Finally, we extracted radiomics features from three types of images: original images, wavelet images, and LoG images. Wavelet images were obtained by applying wavelet transform on the original images. Wavelet transform can decouple informative textures by decomposing the original images into multiple low- and high-frequency components. Let *H* and *L* be a high-pass and low-pass wavelet function, respectively. Then, the eight decomposed images can be denoted as ***I****_HHH_*, ***I****_HHL_*, ***I****_HLH_*, ***I****_HLL_*, ***I****_LHH_*, ***I****_LHL_*, ***I****_LLH_*, ***I****_LLL_*, where the three subscripts meant the high- or low-pass filtering operations along *x*, *y* and *z* directions of the original 3D MR image. LoG images were obtained by applying LoG filtering operation on the original images. LoG performs two filtering operations, a Gaussian filtering and a Laplacian filtering. Finally, 11 features comprising 3 texture features and 8 intensity features were selected for the final radiomics model building. All 11 features were from wavelet images, as shown in **Table 2**. The meanings of these features are described as follows.

| **No.** | **Selected Features** | **Meanings** |
| --- | --- | --- |
| *f*_1_ | Median | The median gray level intensity within the tumor area. |
| *f*_2_ | GLCM. Cluster Shade | A measure of the image uniformity. A higher cluster shade implies greater asymmetry about the mean. |
| *f*_3_ | Mean | The mean gray level intensity within the tumor area. |
| *f*_4_ | Root Mean Squared | The square-root of the mean of all the squared intensity values. It is another measure of the magnitude of the image values. |
| *f*_5_ | GLSZM. Small Area Low Gray Level Emphasis | A measure of the proportion in the image of the joint distribution of smaller size zones with lower gray-level values. |
| *f*_6_ | Median | The median gray level intensity within the tumor area. |
| *f*_7_ | Skewness | Skewness measures the asymmetry of the distribution of values about the Mean value. *f*_7_ is extracted from ***I****_HLL_* of T1c image. |
| *f*_8_ | Maximum | The maximum gray level intensity within the tumor area. |
| *f*_9_ | GLCM. Inverse Variance | A measure of the local homogeneity of an image. |
| *f*_10_ | Skewness | The same as *f*_7_ but extracted from ***I****_LHH_* of FLAIR image. |
| *f*_11_ | Skewness | The same as *f*_7_ but extracted from ***I****_LHL_* of FLAIR image. |
